# Sequestration and efflux largely account for cadmium and copper resistance in the deep‐sea *Nitratiruptor* sp. SB155‐2 (phylum Campylobacterota)

**DOI:** 10.1111/1462-2920.16255

**Published:** 2022-10-28

**Authors:** Ángela Ares, Sanae Sakai, Toshio Sasaki, Shigeru Shimamura, Satoshi Mitarai, Takuro Nunoura

**Affiliations:** ^1^ Marine Biophysics Unit Okinawa Institute of Science and Technology Graduate University Onna Okinawa Japan; ^2^ Super‐Cutting‐Edge Grand and Advanced Research (SUGAR) Program Institute for Extra‐cutting‐edge Science and Technology Avant‐garde Research (X‐STAR), Japan Agency for Marine‐Earth Science & Technology (JAMSTEC) Yokosuka Japan; ^3^ Imaging section, Okinawa Institute of Science and Technology Graduate University Onna Okinawa Japan; ^4^ Research and Development Center for Marine Biosciences Japan Agency for Marine‐Earth Science and Technology (JAMSTEC) Yokosuka Japan

## Abstract

In deep‐sea hydrothermal vent environments, metal‐enriched fluids and sediments abound, making these habitats ideal to study metal resistance in prokaryotes. In this investigation, we employed transcriptomics and shotgun proteomics with scanning transmission electron microscopy and energy‐dispersive x‐ray spectroscopy (STEM‐EDX) to better understand mechanisms of tolerance for cadmium (Cd) and copper (Cu) at stress‐inducing concentrations in *Nitratiruptor* sp. SB155‐2 (phylum Campylobacterota). Transcriptomic profiles were remarkably different in the presence of these two metals, displaying 385 (19%) and 629 (31%) differentially transcribed genes (DTG) in the presence of Cd(II) and Cu(II), respectively, while only 7% of differentially transcribed (DT) genes were shared, with genes for non‐specific metal transporters and genes involved in oxidative stress‐response predominating. Transcriptomic and proteomic analyses confirmed that metal‐specific DT pathways under Cu(II) stress, including those involving sulfur, cysteine, and methionine, are likely required for high‐affinity efflux systems, while flagella formation and chemotaxis were over‐represented under Cd(II) stress. Consistent with these differences, STEM‐EDX analysis revealed that polyphosphate‐like granules (pPLG), the formation of CdS particles, and the periplasmic space are crucial for Cd(II) sequestration. Overall, this study provides new insights regarding metal‐specific adaptations of Campylobacterota to deep‐sea hydrothermal vent environments.

## INTRODUCTION

Bacteria are widely recognized for efficient adaptation to environmental stress, including adaptation to high concentrations of metals. Unlike eukaryotes, bacteria lack compartments to store metal ions; hence, cellular homeostasis relies on metal ion uptake, efflux, and sequestration (Chandrangsu et al., 2017; Ma et al., 2009; Nies, 2003) to regulate net accumulations of metals in the cytoplasm. Metals such copper, manganese, nickel, or zinc are required for biological processes in only trace quantities (Reyes‐Caballero et al., 2011), while others like cadmium, lead, and silver have no known biological function, so it is crucial to remove them from the cytosol immediately. Metal ion uptake and efflux are commonly regulated by metalloregulatory proteins and high‐affinity transporters that respond to various metal ions in highly selective ways. Predominant among efflux systems responsible for copper (Cu)(II) homeostasis are the resistance‐nodulation‐division (RND) protein superfamily *cus* determinant (Franke et al., 2003) and P‐type ATPase CopA, which pumps Cu ions out of the cell (Fan et al., 2001; Outten et al., 2000) in combination with multicopper oxidase CueO (Grass & Rensing, 2001). In contrast, under cadmium (Cd) stress, different systems operate, such as the genetic determinants, *cad* and *czc*, which export excess Cd(II), as well as other ions, like zinc or lead (Ducret et al., 2020; Naz et al., 2005; Nies, 1995; Nucifora et al., 1989). Additionally, sequestration of metals inside or outside the cell constitutes another major strategy. For this purpose, polyphosphate‐like granules (pPLGs), long‐chain anionic polymers of phosphate, constitute a non‐selective strategy employed by many bacteria (Keasling & Hupf, 1996; Villagrasa et al., 2020). Formation and hydrolysis of pPLGs are mainly due to the action of polyphosphate kinases (PPKs) and exopolyphosphatases (PPXs), respectively, and their activities can be modulated by metal concentrations in the cytosol, among other factors (Docampo, 2006). When metal ions are present in the environment, pPLGs can efficiently sequester them, but in some species and with some metals, accumulation of metal ions also tends to stimulate PPX activity, promoting hydrolysis into inorganic phosphate molecules, so that metal–phosphate complexes can be transported out of the cells (Alvarez & Jerez, 2004; Rivero et al., 2018). Another means of reducing metal presence in the cytosol is through biomineralization of metals. A number of bacteria are able to precipitate metal‐laden particles in the periplasmic space. Biomineralization has been observed when bacteria are exposed to a variety of metals. Cd is converted to cadmium sulfide particles (CdS) (Ma et al., 2020; Ma & Sun, 2021; Wang et al., 2017; Yang et al., 2015). Lead is excreted as galena (PbS) or lead(II) phosphate (Pb_3_(PO_4_)_2_) (Zhang et al., 2019,) and Cu(II) is converted to chalcocite (Cu_2_S) (Kimber et al., 2020). Many strategies to mitigate metal stress are metal specific; hence, performing comparative metal studies (Hu et al., 2005; Jiang et al., 2020; Lu et al., 2017; Nies, 1995) in the same experimental set‐up, enables metal‐specific mechanisms to be distinguished from generalized molecular pathways. Furthermore, under natural conditions, various metals co‐occur in the same micro‐habitat, as in deep‐sea hydrothermal vents.

In deep‐sea hydrothermal vent environments, metals and reduced gas‐rich hydrothermal fluids form steep physico‐chemical gradients, and organisms must adapt such extreme environments (Reysenbach et al., 2000; Takai & Nakamura, 2010). In recent decades, several studies have focused on metal resistance strategies in deep‐sea vent chemosynthetic bacteria (Crepo‐Medina et al., 2009; Fukui et al., 2005; Jeanthon & Prieur, 1990; Lagorce et al., 2012; Vetriani et al., 2005).

The phylum Campylobacterota (formerly Epsilonproteobacteria) includes representative primary producers in hydrothermal ecosystems, and in fact, they account for 66%–98% of the microorganisms associated with hydrothermal vent substrates (Lopez‐Garcia et al., 2003; Nakagawa, Takai, Inagaki, Chiba, et al., 2005a; Nakagawa, Takai, Inagaki, Hirayama, et al., 2005b; Takai et al., 2003b; Vetriani et al., 2014). However, to date, adaptive mechanisms that enable Campylobacterota to thrive in metal‐rich environments remain poorly understood. In this study, we characterized molecular responses in combination with morphological adaptations to Cd(II) and Cu(II) stress in the deep‐sea hydrothermal vent bacterium, *Nitratiruptor* sp. SB155‐2. *Nitratiruptor* sp. SB155‐2, is a gram‐negative, chemolithoautotrophic bacterium of the phylum Campylobacterota isolated from the Okinawa Trough. It is capable of growing under microaerobic and anaerobic conditions using hydrogen, elemental sulfur, sulfide, and thiosulfate as electron donors and oxygen and nitrate as electron acceptors (Nakagawa et al., 2007). Genomic features of *Nitratiruptor* sp. SB155‐2 revealed ≥20 responsive genes in a wide variety of metal transport systems, as well as detoxification mechanisms of heavy metals such as arsenic, cadmium, copper, manganese, or zinc (Nakagawa et al., 2007), but these were not studied in detail. Special attention was paid to distinguishing metal‐specific versus generalized strategies to mitigate metal stress. To achieve this, we employed high‐throughput scanning transmission electron microscopy (STEM) coupled with energy‐dispersive x‐ray (EDX) spectroscopy, whole‐transcriptome RNA‐seq analysis and shotgun proteomic analysis.

## EXPERIMENTAL PROCEDURES

### Bacterial strain growth conditions

*Nitratiruptor* sp. SB155‐2 was kindly provided by Satoshi Nakagawa (Kyoto University). This strain was isolated from a 30‐m sulfide mound in the Iheya North field of the Okinawa Trough (27°47′50 N, 126°53′80 E). The depth of collection was 968 m and the maximum temperature of the vent water was 311°C (Takai et al., 2003a). Its physiological and genomic features were characterized previously (Nakagawa et al., 2007; Nakagawa, Takai, Inagaki, Hirayama, et al., 2005b). Strain SB155‐2 was maintained axenically in MMJS artificial seawater (Nunoura et al., 2008) containing NaNO_3_ and NaHCO_3_ (1 g/L) at 55°C and a pH of 6.7, without shaking, as described previously (Nakagawa et al., 2007). Glass bottles (100 ml) (Schott Duran, Germany) containing 30 ml of medium were used throughout the study. To initiate each culture, 0.5 ml of the inoculum (10^7^–10^8^ cells/ml) were added to these bottles. Tubes and bottles were closed with rubber stoppers and headspace gas was replaced with H_2_/CO_2_ (80:20) at a gas pressure of 0.2 MPa.

### Heavy metal treatments

To test metal tolerance, *Nitratiruptor* SB155‐B responses to Cd(II) and Cu(II) were evaluated at different concentrations (from 0.01 to 0.5 mM). Stock solutions of these elements were prepared by dissolving the respective salt (CdCl_2_・2.5 H_2_O or CuSO_4_・5 H_2_O) (FUJIFILM Wako, Japan) in MilliQ water. To prepare working metal solutions, required volumes were added to the cultures by filtration through 0.22‐μm membrane filters (Millipore Corp., USA).

#### Growth measurements

Cell growth was studied under different treatments (Cd: 0.05, 0.1 and 0.5 mM; Cu: 0.01, 0.05 and 0.1 mM, *n* = 4) and control cultures in order to determine appropriate sublethal concentrations. Metal concentrations applied in this study were selected based on a previous large‐scale screening by testing different concentration from 0.01 to 10 mM and evaluating bacteria proliferation in detail. An aliquot of 1 ml was collected every 12 h for 5 days. Cell densities were determined using standard flow cytometry (Brussaard, 2004; De Corte et al., 2012) with minor modifications. Briefly, sample aliquots were fixed with glutaraldehyde (0.5% final concentration) and frozen at −80°C until quantification. After thawing on ice, samples were stained with SYBR Green I (Molecular Probes, Invitrogen, Carlsbad, USA). Cell numbers were counted using an Accuri C6 flow cytometer (BD Biosciences, US) and fluorescence versus side scatter was plotted.

#### Incubation for STEM‐EDX and RNA‐seq analysis

Once cultures reached late‐exponential phase, that is, after 4 days, metals were included at a final concentration of 0.01 mM for Cd(II) and 0.05 mM for Cu(II). Bottles were incubated for 24 h (*n* = 3) for microscopy and 3 h for RNA‐seq analysis (*n* = 4). After incubation, samples were immediately used for STEM‐EDX preparation or rapidly filtered through 0.2‐μm PTFE filters (Merck, Germany), flash frozen in liquid nitrogen, and stored at −80°C until RNA extraction.

### Electron microscopy observation

#### STEM‐EDX observation

After metal incubation, *Nitratiruptor* sp. SB155B cells were collected by centrifugation at 4000 rpm for 5 min at 4°C. Fixation was carried out in 2.5% (v/v) glutaraldehyde in 0.1 M sodium cacodylate buffer for 2 h in darkness at room temperature. Then, supernatants were removed and samples were post‐fixed with osmium tetroxide (OsO_4_) for 1 h. Samples were dehydrated in an ethanol series (70%, 80%, 90%, 95% and 100%) and finally embedded in resin. Fixed samples were cut using a Leica UC6 microtome (Leica, Germany) with a diamond cutter (DiATOME, US) at a thickness of 100 nm. Finally, sections mounted on carbon‐coated nickel grids (Nisshin‐EM, Japan) were examined by STEM with a JEOL ARM‐200F system at 80 Kv with an angular annular dark field detector (30–120 mrad) using HR STEM‐HAADF observation mode. The spot size was 6C for observing and 1C for EDX mapping.

For EDX detection, a JEOL SDD high‐resolution EDX detector (100 mm^2^ solid angle) was used. Elemental spectra were collected from at least two points per cell and a minimum of 30 cells per treatment. For characterization, total cell composition was mapped in at least at five cells per treatment for 5–15 h. Atomic composition of ratios and distribution were assessed using the software, JED‐2300 Analysis Station.

#### TEM observation of flagella

In order to examine whether Cd‐treated cultures produced larger proportions of flagella, negative staining evaluation was performed with TEM. Cells from Cd‐treated and control samples (*n* = 3) were gently collected by centrifugation at 2000 rpm for 4 min at 4°C, in order to not disrupt flagella, and a drop was collected and directly mounted on a carbon‐coated nickel grid. Samples were negatively stained with 1% uranyl acetate and observed under a JEM1230R electron microscope at an accelerating voltage of 100 keV. More than 200 cells were observed for presence/absence of flagella.

### Total RNA isolation, library preparation, and sequencing

Total RNA was extracted from filters using ZR Fungal/Bacterial RNA kits (Zymo Research, USA) following manufacturer instructions. Samples were treated with DNase I (Qiagen, Germany) for DNA removal. Following extraction, RNA quality and concentration were assessed on an Agilent 2100 Bioanalyzer and a Qubit 2.0 fluorometer, respectively. After quality was determined, two replicates for Cd(II) and Cu(II) treatments and three replicates for controls met the quality criteria to proceed with subsequent steps. Total rRNA was extracted from samples using Ribo‐zero Magnetic kits (Illumina, USA). For library preparation, NEBNext® Ultra Directional RNA Library Prep Kits for Illumina® (Illumina) were used following manufacturer instructions. Quality control of prepared libraries was determined with an Agilent 2100 Bioanalyzer, and after library normalization, cDNA libraries were sequenced on an Illumina NovaSeqTM 6000 sequencing system with a 2 × 150‐bp pair‐end read length protocol.

### RNA‐seq data analyses

The resulting FASTA files were processed using the Nextflow pipeline nfcore/rnaseq (version 3.1) mainly with standard settings (Ewels et al., 2020). However, the few changes made to the settings are summarized as follows: (i) strandedness of the library was set as reverse in the input file; (ii) Hisat2 was the aligner selected; (iii) in order to remove adaptors and low‐quality sequences, the Trim Galore clipped length was changed to 15 bp. Reads were mapped to the reference sequence *Nitratiruptor* sp. SB155‐2 (GenBank: Assembly: GCA_000010325.1). Gene counts for each sample were extracted from StringTie results using the python script, prepDE.py and imported into the R statistical environment for further analysis. In order to identify potential outliers and major sources of variation, hierarchical clustering heatmap and principal component analysis were performed after RLD normalization. Differential gene expression analysis between metal‐treated cultures was performed with the DESeq function in the Bioconductor package, DESeq2 (Love et al., 2014). Genes that were considered differentially transcribed with a false discovery rate (FDR) adjusted *p* value (padj) less than 0.05 and a change of at least 2‐fold (log2FoldChange = 1) were considered statistically significant.

Gene Ontology (GO) information was obtained using Blast2Go software (version 5.2.5). Identification of GO terms enriched among differentially transcribed genes was carried out with the hypergeometric test in the R package, GOstats (Falcon & Gentleman, 2007). Differentially enriched GO terms were visualized in semantic similarity‐based scatterplots using REVIGO (http://revigo.irb.hr/). Identification of enriched KEGG pathways was further investigated by applying the Kegga function in the R package, edgeR (Robinson et al., 2010). Both GO terms and KEGG pathways were considered significantly enriched with a *p* value less than 0.05. Sequencing data have been deposited in the NCBI Sequencing Read Archive under accession PRJNA746661.

### Quantitative PCR

To confirm RNA‐seq data, expression levels of 11 representative genes differentially transcribed in samples growing under both Cu(II) and Cd(II) stress were examined by quantitative PCR (qPCR). Names of the selected genes, as well as the primers used for qPCR, can be found in Table S7. After extraction of RNA, cDNA was synthesized from three replicates per treatment using SuperScript IV VILO Master Mix (Invitrogen, USA). All (qRT‐PCR) reactions were carried out with iQ SYBR Green Supermix (Bio‐Rad, USA) on a StepOnePlus real‐time PCR system (ThermoFishcher Scientific) using the following conditions: an initiation step at 95°C for 3 min followed by 40 cycles of PCR amplification at 95°C for 15 s and 60°C for 30 s. The cell division protein FtsZ (FtsZ, NIS_RS05555) was used as the reference gene based on previous studies (Rocha et al., 2015) and relative gene expression levels were calculated using the 2−ΔΔCT method (Livak & Schmittgen, 2001). No template controls were used for target genes in order to control non‐targeted amplification.

### Shotgun proteomics

Following RNA‐seq analysis, *Nitratiruptor* sp. SB155‐2 cells harvested at late‐exponential phase were used for semi‐quantitative shotgun proteomics. Cells suspended in 200 μl of cell lysis buffer (100 mM triethylammonium bicarbonate [TEAB], pH 8.6; 2 mM phenylmethylsulfonyl fluoride [PMSF]) were disrupted by sonication. The protein concentration of the cell‐free extract was determined using Qubit Protein assay kit (Thermo Fisher Scientific).

The cell‐free extract with 10 μg protein was suspended in 20 μl denaturing buffer MPEX PTS Reagents Sol A + B (GL Science) and incubated at 95°C for 5 min. Then the solution was sonicated for 10 min using an ultrasonic bath (LEO‐80; Tokyo Garasu Kikai, Japan), for solubilization. After adding 1 μl of 500 mM DTT (Thermo Fisher Scientific), the denatured protein was incubated at 95°C for 5 min and left at room temperature for 25 min. For alkylation, 1 μl of 500 mM iodoacetamide (Thermo Fisher Scientific) was added, and the solution was incubated at room temperature for 30 min in the dark.

Tryptic digestion was performed at 37°C for 3 h after additions of 76 μl of MPEX PTS Reagents Sol A and 1 μl of 100 ng Trypsin Protease (Thermo Fisher Scientific). Subsequently, 1 μl of MS grade Trypsin Protease was added again and proteins were further digested at 30°C overnight. Tryptic peptides were purified with liquid–liquid extraction using MPEX PTS Reagents Sol C and D, according to the manufacturer's instructions. Furthermore, residual surfactants were removed using Pierce Detergent Removal Spin Columns (Thermo Fisher Scientific). Then, dried peptides were suspended with 2% acetonitrile/0.1% trifluoroacetic acid solution, and were subjected to LC–MS/MS analysis.

Data acquisition and analysis were performed as described previously (Nunoura et al., 2008), with a modification using label‐free quantification method in Proteome Discoverer 2.2 software package (Thermo Fisher Scientific) to obtain semi‐quantitative data set. Proteins that were considered differentially more abundant with an adjusted *p* value (padj) less than 0.05 and a change of at least 2‐fold were considered statistically significant.

## RESULTS

### Effect of sublethal metal concentrations on growth of *Nitratiruptor* sp. SB155‐2

**FIGURE 1:**
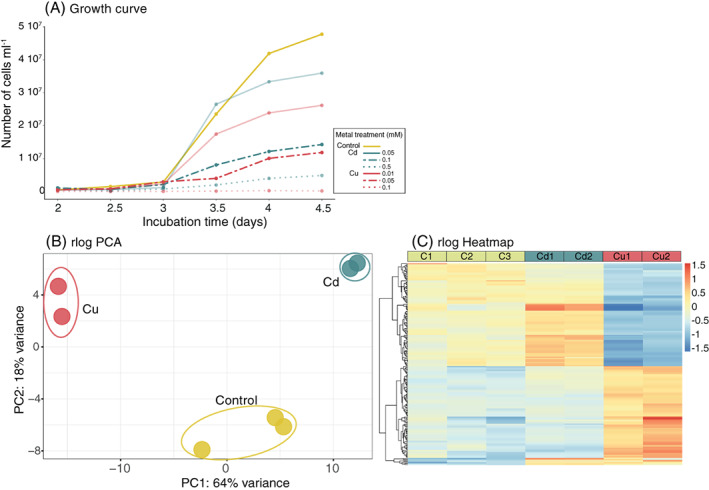
Growth performance of *Nitratiruptor* sp. SB155‐2 cells affected by different metal concentrations and PCA and heatmap analysis of RNA‐seq transcriptome data demonstrated metal‐specific gene expression patterns. (A) Aliquots of 0.1 ml were collected from each bottle every 12 h for 5 days, stained with SYBR Green I and quantified using an Accuri C6 flow cytometer. Values are means of four replicates. Sub‐lethal concentrations at which subsequent RNA‐seq analysis and microscopy observations were performed are indicated with 100% opacity in the corresponding control, Cd 0.1 mM, and Cu(II) 0.05 mM. (B) A PCA plot of all RNA‐seq samples from regularized, log transformed counts (rlog) showing how samples subjected to different treatments clustered separately. (C) A heatmap including 300 genes with the lowest false discovery rate (FDR) adjusted *p* values. Cell colour represents differences from the mean regularized log transformed count for each contig in each sample. Colour code is indicated in the figure legend. C = controls; Cd = CdCl_2_ 0.1 mM; Cu = CuSO_4_ 0.05 mM

Growth of *Nitratiruptor* sp. SB155‐2 was monitored for 5 days by measuring cell densities every 12 h in the presence of cadmium (Cd) (0.05, 0.1, and 0.5 mM), or copper (Cu) (0.01, 0.05, and 0.1 mM), compared with controls (Figure 1A). Cd(II) and Cu(II) were selected because both are found in Okinawa Trough deposits (Chen et al., 2000) and are well known for their high toxicity for various microbial groups, which make them model elements for microbial toxicology studies (Arguello et al., 2013; Hu et al., 2005; Nies, 2003). Additionally, these elements have different biological roles. Whereas Cu(II) is essential as a redox cofactor in catalytic centres of various enzymes (Cobine et al., 2006; Gort et al., 1999), Cd(II) serves no known function in living organisms.

In order to select meaningful stress conditions, the objective was to find the highest metal concentration that did not inhibit cell growth. For both elements, the medium concentration tested (0.1 mM Cd(II) and 0.05 mM Cu(II)) impacted but did not inhibit cell growth completely (Figure 1A), while allowing cell densities sufficient to perform downstream analyses.

### Overview of RNA seq results

Illumina NovaSeqTM 6000 sequencing produced over 37.8 million read pairs with the number of read pairs per sample ranging from 4.9 to 6.6 million. After trimming, 37.4 million read pairs (~99%) remained. Transcriptomic coverage of the *Nitratiruptor* sp. SB155‐2 genome varied between 77.9% and 94% (Table S1).

To determine whether various gene transcription profiles reflected different treatments, principal component analysis (PCA) was performed (Figure 1B). Sample variability was higher between experimental treatments than between biological replicates and important differences were found between samples treated with Cd(II) and Cu(II), with replicates clustering well separated along the main axis (64% of total variance). Overall, expression patterns were evaluated using an expression heatmap (Figure 1C), which showed the most significant DE genes ordered by FDR‐adjusted *p* value (*p*adj) for the first 200 genes. The resulting heatmap clearly illustrates different expression profiles in Cu‐treated samples, compared with controls and Cd‐treated samples.

### Differentially transcribed genes

**FIGURE 2:**
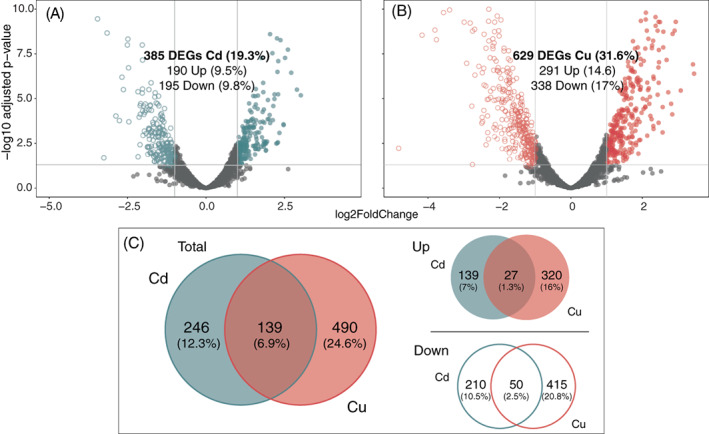
Metal‐specific gene expression pattern in *Nitratiruptor* sp. SB155‐2 with copper induced a significantly higher number of differentially transcribed genes (DTGs). (A, B) volcano plots displaying gene transcription patterns of *Nitratiruptor* sp. SB155‐2 cells treated with cadmium (A) and copper (B) relative to controls. Significantly differentially transcribed genes (DTGs) are highlighted in blue for Cd(II) and red for Cu(II). (C) Venn diagram showing DEGs occurring in both treatments. Log2FoldChange > 1 or <−1, *p*adj ≤ 0.05. C = controls; Cd = CdCl_2_ 0.1 mM; Cu = CuSO_4_ 0.05 mM. Values are means of two replicates.

Overall, the DESeq2 test identified 385 differentially transcribed genes (DTGs) (19.3%) under Cd(II) stress—with 190 and 195 genes significantly up‐ and down‐regulated, respectively (Figure 2A). A more distinct response was found in samples treated with Cu(II), resulting in 31.6% of all genes differentially transcribed (629 genes), 291 up‐regulated and 338 down‐regulated (Figure 2B). Accordingly, DTGs encoding transporter systems, as well as oxidative stress‐responsive genes were found in higher numbers following Cu(II) than Cd(II) stress (Tables S3 and S4). For example, several genes encoding different efflux RND transporter subunits, ABC transporter ATP‐binding protein, oxidoreductases, or thioredoxins/glutaredoxins were specifically up‐regulated following Cd(II) or Cu(II) exposure.

Importantly, of the 139 DT genes that responded to both metals, only 77 were transcribed with the same pattern (27 up‐ and 50 down‐regulated), further demonstrating important differences in the *Nitratiruptor* sp. SB155‐2 metal stress response resulting from exposure to Cd(II) and Cu(II) (Table 1, Figure 2C, Table S2). Table 1 shows the 27 up‐regulated genes that responded to both Cd(II) and Cu(II) stress, corresponding to 1.3% of the total genome. Two groups of contiguous genes were clustered at two different chromosome locations, likely corresponding to two multicomponent systems to transport both Cd(II) and Cu(II) ions from the cytoplasm to the extracellular environment. The first group, NIS_RS00145, NIS_RS00150, and NIS_RS00155, is formed by a metalloregulator ArsR/SmtB family transcription factor, a permease, and an arsenate reductase, ArsC, likely encoding the *ars* operon (Ben Fekih et al., 2018). Other genes likely involved in the *ars* operon, NIS_RS00140 and NIS_RS00160, were up‐regulated following Cu(II) and Cd(II) exposure, respectively. Our BLAST searches showed that the most likely candidates for these genes are arsB and arsR, respectively, according to their genomic location and sequence similarity when compared to *E. coli* (strain K12) (E values 1e^−136^ and 2e^−16^, respectively).

**TABLE 1:** Differentially up‐regulated genes of *Nitratiruptor sp.* SB155‐2 that responded to both cadmium (0.1 mM) and copper (0.05 mM) stress, showing the stress response and expression ratios (in log2FoldChange).

	Gene ID	log2FoldChange		Gene name	Annotation
		Cd	Cu		
1	NIS_RS00145	2.26	2.17		Permease
2	NIS_RS00150	2.14	1.76		Thioredoxin family protein
3	NIS_RS00155	2.13	1.38	*arsC*	Arsenate reductase ArsC
4	NIS_RS00350	2.3	1.17		Long‐chain fatty acid transport protein
5	NIS_RS00380	1.06	1.07	*tetR/acrR*	TetR/AcrR family transcriptional regulator
6	NIS_RS01820	1.59	1.17		Type II secretion system protein
7	NIS_RS02805	1.76	2.18	*groEL*	Chaperonin GroEL
8	NIS_RS02810	2.3	1.4	*groES*	Co‐chaperone GroES
9	NIS_RS04835	1.03	1.29		ATP‐grasp domain‐containing protein
10	NIS_RS04910	3.35	2.46		SO_0444 family Cu/Zn efflux transporter
11	NIS_RS04915	4.34	2.43		Cytochrome c
12	NIS_RS04920	4.47	1.06		Winged helix‐turn‐helix transcriptional regulator
13	NIS_RS04930	3.7	1.43		Hypothetical protein
14	NIS_RS04935	2.18	1.01	*tolC*	Outer membrane protein TolC
15	NIS_RS06020	1.56	1.79	*dnaK*	Molecular chaperone DnaK
16	NIS_RS06025	2.37	1.18	*grpE*	nucleotide exchange factor GrpE
17	NIS_RS06590	1.07	2.3	*proB*	Glutamate 5‐kinase
18	NIS_RS08435	2.03	1.55	*petA*	Ubiquinol‐cytochrome c reductase iron–sulfur subunit
19	NIS_RS08655	1.08	1.01		Alanine‐‐glyoxylate aminotransferase family protein
20	NIS_RS08810	1.56	2.95	*eno*	Phosphopyruvate hydratase
21	NIS_RS08815	1.3	1.37	*recA*	Recombinase RecA
22	NIS_RS09120	3.19	2.94	*degQ*	DegQ family serine endoprotease
23	NIS_RS09125	1.25	1.44		Response regulator transcription factor
24	NIS_RS09510	1.36	1.62	*nosZ*	Sec‐dependent nitrous‐oxide reductase
25	NIS_RS09540	1.09	1.72		c‐type cytochrome
26	NIS_RS09925	2.04	1.53		Type II secretion system protein
27	NIS_RS10305	1.59	1.22		Hypothetical protein

The second cluster encompasses several genes induced under Cd(II) and Cu(II) stress (NIS_RS04910, NIS_RS04915, NIS_RS04920, NIS_RS04930, and NIS_RS04935) and six under Cd(II) stress alone, with NIS_RS04925 likely encoding a multidrug efflux RND transporter. Genes forming this second system encode a SO_0444 family Cu/Zn efflux transporter, a cytochrome, and a TolC family protein. Genes displaying the highest log2FoldChange values for both elements indicated that the SO_0444 family Cu/Zn efflux transporter seems to be central to Cd(II) and Cu(II) detoxification by *Nitratiruptor* sp. SB155‐2.

Other operons commonly up‐regulated included parts of the oxidative stress response, such as the GroES/GroEL or DnaK/DnaJ molecular chaperone systems, glutamate 5‐kinase, or DegQ family serine endoprotease. However, numerous differentially transcribed genes involved in metal transport and oxidative stress differ significantly depending on the metal. As for metal transporters, while only two ABC transporter permeases were differentially transcribed exclusively upon exposure to Cd(II), a larger number of transport‐related genes were differentially transcribed under Cu(II) treatment (Table S3), some of them highly Cu specific. As an example of the latter, the results suggest that both CueO and a P‐type ATPase, with sequence analysis demonstrating a great similarity to the *E. coli* CopA, are up‐regulated exclusively in cultures treated with Cu(II). The combination of these two genes could be an effective mechanism for Cu(II) detoxification in *Nitratiruptor sp*. SB155‐2 (Table S3 and S4).

### Enrichment analysis: GO and KEGG pathways

**FIGURE 3:**
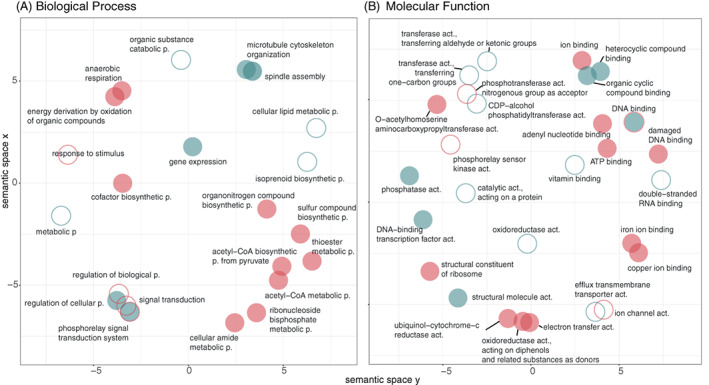
Gene Ontology (GO) enrichment analysis revealed metal‐specific, over‐represented terms ordered by semantic similarities in *Nitratiruptor* sp. SB155‐2 cells when treated with cadmium or copper, relative to controls. A multidimensional scatterplot was created using the tool REVIGO (http://revigo.irb.hr/) and results were exported to the R environment, where they were plotted using the package, ggplot2. The following settings were used: semantic similarity: 0.7 (small), semantic similarity measure: SimRel. Open circles indicate GO terms associated with down‐regulated genes while solid circles indicate up‐regulation for *Biological Processes* (A) and *Molecular Function* (B). Cd treatment is represented in blue while copper is represented in red.

The bioinformatic platform, Blast2GO, was used to annotate gene ontology terms associated with DEGs found in this study. From these, in order to identify over‐represented GO terms involved in the *Molecular Function* (MF) and *Biological Process* (BP) categories under different metal treatments, the R package GOstats, was used (*p*adj < 0.05). Overall, 25 BP GO terms, 9 and 16, in the presence of Cd(II) and Cu(II), respectively, and 29 MF GO terms, 19 and 10, in the presence of Cd(II) and Cu(II), respectively, were over‐represented. BP and MF GO terms were displayed using the REVIGO online tool for effective visualization in a multidimensional scaling plot ordered by semantic similarity (Supek et al., 2011) (Figure 3). Following Cu(II) exposure, GO terms involving *ion* channel, ion binding, sulfur compound biosynthesis, thioester metabolic process, and *O‐acetylhomoserine aminocarboxypropyltransferase* activity terms were over‐represented. In contrast, following Cd(II) exposure, other GO terms were over‐represented, including microtubule cytoskeleton organization, spindle assembly, gene expression, phosphorelay signal transduction system, DNA binding, organic cyclic compound binding or phosphatase activity.

In order to understand which KEGG pathways were enriched among DEGs, the hypergeometric test, Kegga, was used. A total of 16 and 12 signalling pathways were enriched following Cd(II) and Cu(II) exposure, respectively (*p* > 0.05) (Table S6). *Flagellar assembly*, *ribosome*, *RNA degradation* and *bacterial chemotaxis* were the pathways enriched among up‐regulated gene sets following Cd(II) exposure. The dominance of flagella formation in Cd(II)‐treated samples versus controls was further examined by negative staining using TEM. While the proportion of flagella found in control samples accounted for approximately half of the cells, in Cd(II)‐treated samples more than 75% of the cells possessed flagella (Figure S1). Both transcriptomic evidence and negative staining confirmed Cd‐induced formation of flagella in *Nitratiruptor* sp. SB155‐2 cells (Figure S1). As a result of Cu(II) exposure, pathways related to ribosome, microbial metabolism in diverse environments, sulfur metabolism, RNA degradation, nitrogen metabolism and oxidative phosphorylation were over‐represented. The log2FoldChanges of DEGs corresponding to some over‐represented KEGG pathways are summarized in Table 2. Importantly, both KEGG and GO enrichment analyses revealed that genes involved in sulfur, methionine, and cysteine metabolism pathways are over‐represented among up‐regulated genes of *Nitratiruptor* sp. SB155‐2 treated with Cu(II) (Table 2, Figure 3, Tables S5 and S6). Among genes involved in sulfur metabolism, glutathione synthase was exclusively up‐regulated in Cu‐treated samples (Table S4) suggesting that glutathione serves a fundamental function in Cu(II) stress.

**TABLE 2:** Differentially transcribed genes involved in (A) sulfur metabolism, (B) cysteine and methionine and (C) chemotaxis and flagella formation and expression ratios (in log2FoldChange) of *Nitratiruptor* sp. SB155‐2 cells following cadmium (0.1 mM) or copper (0.05 mM) exposure

	Gene ID	log2FoldChange		Gene name	Annotation
		Cd	Cu		
(A) Sulfur metabolism					
1	NIS_RS00170		1.82		NAD(P)/FAD‐dependent oxidoreductase
2	NIS_RS00180		2.31	*soxY*	Thiosulfate oxidation carrier protein SoxY
3	NIS_RS00185		1.57	*soxZ*	Thiosulfate oxidation carrier complex protein SoxZ
4	NIS_RS00795		2.45		FAD‐dependent oxidoreductase
5	NIS_RS00855		1.84		NAD(P)/FAD‐dependent oxidoreductase
6	NIS_RS01780		1.48		FAD‐dependent oxidoreductase
7	NIS_RS02255		1.9		Bifunctional oligoribonuclease/PAP phosphatase NrnA
8	NIS_RS09715		1.72	*soxB*	Thiosulfohydrolase SoxB
9	NIS_RS09720		1.94	*soxA*	Sulfur oxidation c‐type cytochrome SoxA
10	NIS_RS09725		1.39	*soxZ*	Thiosulfate oxidation carrier complex protein SoxZ
(B) Cysteine and methionine					
1	NIS_RS03035		1.38	*metH*	Methionine synthase
2	NIS_RS06840		2.27		O‐acetylhomoserine aminocarboxypropyltransferase/cysteine synthase
3	NIS_RS06845	0.86	2.1		O‐acetylhomoserine aminocarboxypropyltransferase/cysteine synthase
4	NIS_RS08135		1.24		S‐adenosylmethionine decarboxylase proenzyme
5	NIS_RS08270		1.38		Homoserine dehydrogenase
6	NIS_RS08520		1.49	*luxS*	S‐ribosylhomocysteine lyase
C) Chemotaxis and flagella formation					
1	NIS_RS01505	1.07		*cheB*	Chemotaxis‐specific protein‐glutamate methyltransferase CheB
2	NIS_RS03445	1.12		*motB*	Flagellar motor protein MotB
3	NIS_RS03500	1.23		*motB*	Flagellar motor protein MotB
4	NIS_RS05300	1.1			MotA/TolQ/ExbB proton channel family protein
5	NIS_RS05305	1.34			Flagellar basal body‐associated FliL family protein
6	NIS_RS05310	1.2			Flagellin
7	NIS_RS09980	1.4		*flgL*	Flagellar hook‐associated protein FlgL

### Gene expression validation by qPCR

**FIGURE 4:**
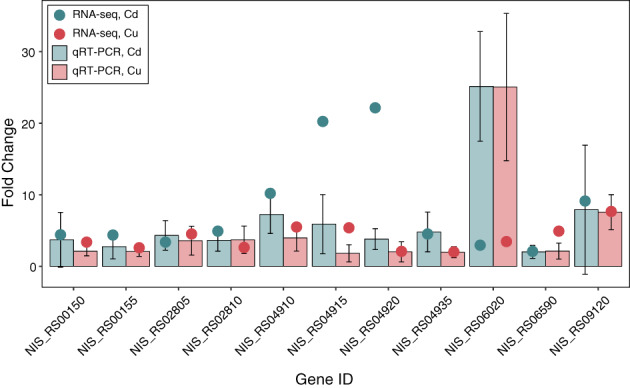
Validation of RNA‐Seq data by real‐time qRT‐PCR. Eleven genes of *Nitratiruptor* sp. SB155‐2 commonly up‐regulated under 3‐h treatment with Cd or Cu(II) were chosen to validate RNA‐Seq data by qPCR. Bars and points represent mean fold changes obtained for three biological qPCR and RNA‐seq replicates, with error bars showing standard deviations. Blue bars and points represent data for qRT‐PCR and RNA‐seq, respectively. Values are means of three replicates. Gen IDs annotations: NIS_RS00150, thioredoxin family protein; NIS_RS00155, arsenate reductase ArsC; NIS_RS02805, chaperonin GroEL; NIS_RS02810, co‐chaperone GroES; NIS_RS04910, SO_0444 family Cu/Zn efflux transporter; NIS_RS04915, cytochrome c; NIS_RS04920, winged helix‐turn‐helix transcriptional regulator; NIS_RS04935, outer membrane protein TolC; NIS_RS06020, molecular chaperone DnaK; NIS_RS06590, glutamate 5‐kinase; NIS_RS09120, DegQ family serine endoprotease. Colour code is indicated in the figure legend.

Quantitative PCR was performed on the following 11 up‐regulated genes among those found responsive to both metals (Table 1): NIS_RS00150, thioredoxin family protein; NIS_RS00155, arsenate reductase ArsC; NIS_RS02805, chaperonin GroEL; NIS_RS02810, co‐chaperone GroES; NIS_RS04910, SO_0444 family Cu/Zn efflux transporter; NIS_RS04915, cytochrome c; NIS_RS04920, winged helix‐turn‐helix transcriptional regulator; NIS_RS04935, Outer membrane protein TolC; NIS_RS06020, molecular chaperone DnaK; NIS_RS06590, glutamate 5‐kinase; NIS_RS09120, DegQ family serine endoprotease. Overall, the selected genes were more transcribed in metal‐treated samples than controls, and with similar values obtained by RNA‐Seq (Figure 4). Only a few of them showed significant differences in expression levels between the two techniques. For instance, levels of expression of NIS_RS04910 and NIS_RS04915 in samples treated with Cd(II) were significantly higher with RNA‐seq than qPCR analysis. Contrary to this pattern, expression levels of NIS_RS06020 were significantly higher in qPCR than RNA‐seq analysis under both Cd(II) and Cu(II) stress (Figure 4). In general, these results suggest that the expression patterns from RNA‐seq analysis are reliable.

### Shotgun proteomics

**FIGURE 5:**
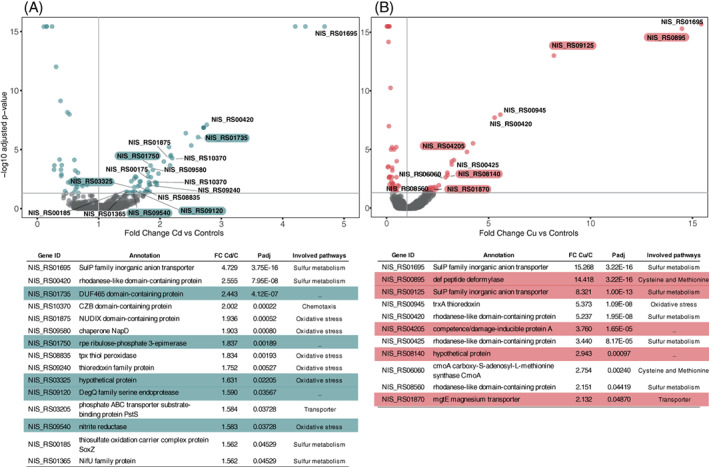
Shotgun proteomic analysis of *Nitratiruptor* sp. SB155‐2 when treated with metals showed high expression levels of proteins involved in sulfur metabolism, and cysteine, methionine, and chemotaxis pathways. Volcano plots displaying quantified differentially expressed proteins of *Nitratiruptor* sp. SB155‐2 cells treated with cadmium (A) and copper (B) relative to controls. Genes differentially overexpressed in both RNA‐seq and proteomics analysis are highlighted in the figure and the corresponding table in blue and red for cadmium and copper treatment, respectively. Proteins presented were those previously identified by enrichment analysis. Values are means of three replicates. Grey lines indicate thresholds of *p* value and fold change.

In order to confirm genetic patterns observed with RNA‐seq, semi‐quantitative shotgun proteomics were also performed. This analysis allowed us to provide a more complete picture of molecular mechanisms underlying metal stress in *Nitratiruptor* sp. SB155‐2. Shotgun proteomics identified 98 proteins differentially abundant under Cd(II) stress, with 49 of them significantly up‐regulated (Figure 5A). A slightly less distinct response was found in samples treated with Cu(II), resulting in 94 of all total quantified proteins differentially transcribed, only 29 of the genes up‐regulated (Figure 5B). For each treatment, only five genes were up‐regulated in common with RNA‐seq analysis data (Figure 5). Some of these common genes were implicated in oxidative stress in the case of Cd(II) (NIS_RS03325, NIS_RS09540), sulfur metabolism, cysteine and methionine pathways, and transport in the case of Cu(II) (NIS_RS09125, NIS_RS00895, and NIS_RS01870). Accordingly, a number of the quantified proteins are involved in the main pathways and other groups of genes identified by RNA‐seq in both treatments. For instance, chemotaxis in the case of Cd(II) and cysteine and methionine in the case of Cu(II), while proteins involved in sulfur metabolism and oxidative stress response were present in higher proportions under both treatments (Figure 5).

### STEM observation and presence of pPLGs granules

**FIGURE 6:**
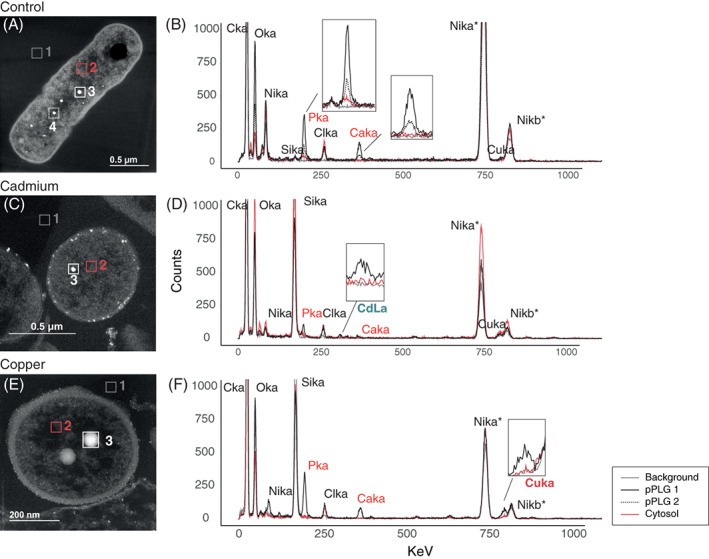
Scanning transmission electron microscopy (STEM) micrographs demonstrated the presence of polyphosphate‐like granules (pPLGs) under all treatments (A. control, B. cadmium 0.1 mM and C. copper 0.05 mM) while energy dispersive spectroscopy (EDX) analysis showed that pPLG elemental composition is metal‐specific in *Nitratiruptor* sp. SB155‐2. In each STEM micrograph, a square area mapping intracellular polyphosphate‐like granules (pPLG), cytosol, and background was selected for elemental composition analysis and the resulting spectra are shown for the various treatments. Line colour and style represent different mapping locations in the cell. Ten cells per treatment were analysed and produced similar spectral patterns. Colour code is indicated in the figure legend; * indicates that the high number of counts of Ni comes from the Ni grid supporting the sample.

**FIGURE 7:**
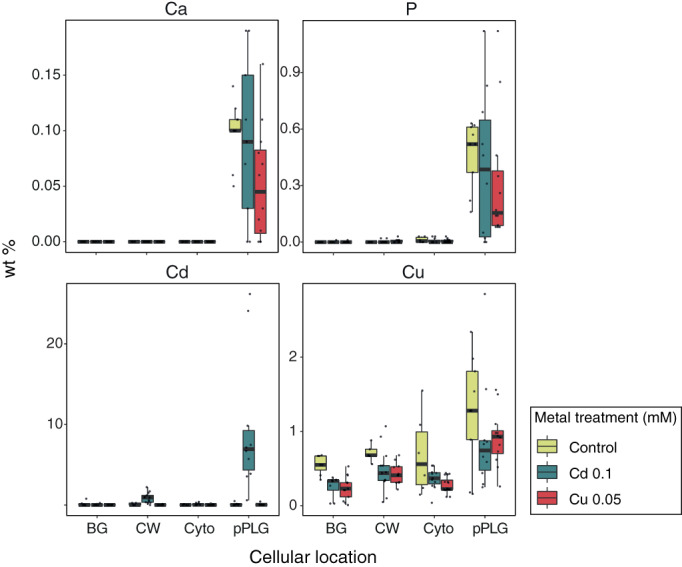
Cadmium treatment, but not copper, caused metal enrichment in polyphosphate‐like granules in *Nitratiruptor* sp. SB155‐2 cultures. Relative concentrations were compared at different cellular locations in *Nitratiruptor* sp. SB155‐2 cells cultured in MMJS artificial seawater control (yellow) or with addition of Cd (0.1 mM) (blue) and Cu(II) (0.05 mM) (red) for 24 h. Concentrations were estimated by area mapping using energy dispersive spectroscopy (EDX) and are shown in weight percent of dry mass (% wt). Ten individual cells per treatment were analysed as replicates. Colour code is indicated in the figure legend. BG, background; CW, cell wall; Cyto, cytoplasm; pPLG, polyphosphate‐like granules

*Nitratiruptor* sp. B155‐2 thin sections were observed using transmission electron microscopy in scanning mode (STEM) to investigate morphological differences under metal treatments versus controls. No visible differences in cell morphology were observed in regard to the cytosol or cell wall (Figure S3). Round, electron‐dense granules in the cytoplasm were confirmed under all treatments (Figure S3 and S4) with moderately different numbers and sizes. These granules were somewhat more abundant in Cd‐treated cultures than in controls, while Cu(II) seemed to trigger formation of more and relatively larger granules per cell (Figure S4). Figure 6 shows the spectral profiles resulting from EDX spectroscopy square mapping at different locations (cell cytosol, matrix background and granules) of cells cultured with and without metal. Chemical analysis of intracellular granules confirmed enrichment with phosphorus and calcium, identifying them as polyphosphate‐like granules (pPLGs) (Figure S2, Figures 6 and 7). Relative composition analysis by EDX confirmed that pPLG phosphorus and calcium levels were similar under all treatments tested (Figure 7).

### Metal localization by EDX analysis

**FIGURE 8:**
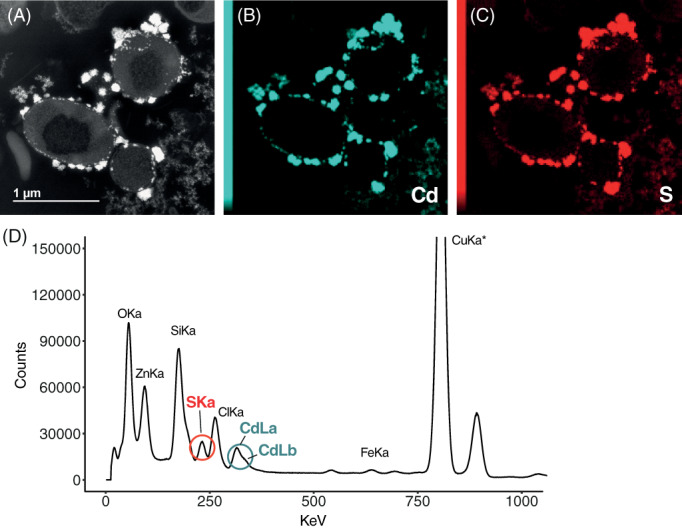
Cadmium treatment (0.1 mM, 24 h) induced formation of CdS particles attached to the outer cell membrane in *Nitratiruptor* sp. SB155‐2 cells. The 15‐h energy dispersive spectroscopy (EDX) mapping was carried out to determine the elemental composition of extracellular particles found in Cd‐treated samples. Panel (A–C) shows the STEM micrograph, Cd and S localization in the area, respectively. (D) Shows the energy dispersive spectrum analysis of the whole micrograph, indicating where the peaks for Cd and S are located; * indicates that a high number of counts of Cu come from the Cu grid supporting the sample.

EDX analysis revealed the presence of Cd(II) in pPLGs of cells grown under Cd(II) exposure (Figures 6 and 7, Figure S2). No Cd(II) was detected in pPLGs of control cells or in those treated with Cu(II). Cd(II) concentrations were higher in pPLG granules than in the cell wall, which also showed Cd‐enriched particles (Figures 6 and 7 and Figure S2). In contrast, measurable levels of Cu(II) were found at different cell locations under all treatments tested, that is, cytosol, cell wall, matrix background, and pPLG. No significant differences in Cu(II) content were found in pPLGs between treatments, because concentrations were highly variable. Additionally, using STEM, electron‐dense particles were also observed attached to the outer membrane surface of cells following Cd(II) exposure. Long‐term mapping with EDX confirmed that the main elements included were Cd(II) and S (Figure 8). In addition to pPLGs and CdS, high‐resolution STEM enabled identification of Cd(II) particles at the periphery of the cells (Figure 7), likely located in the periplasmic space.

## DISCUSSION

The Campylobacterota is one of the dominant bacterial taxa inhabiting deep‐sea hydrothermal vent ecosystems. A large body of literature has documented their contributions to vent biogeochemical processes, such as metabolic pathways to fix inorganic carbon, reduce nitrogen, or unique sulfur metabolism (Akerman et al., 2013; Campbell et al., 2006; Vetriani et al., 2014; Yamamoto & Takai, 2011). Nonetheless, adaptations to their metal‐rich niche remain little studied. This investigation examined whole genomic, transcriptomic, and proteomic profiles of the deep‐sea hydrothermal vent bacterium, *Nitratiruptor* sp. SB155‐2 (phylum Campylobacterota), following exposure to Cd(II) and Cu(II). In addition, high‐throughput microscopy was applied to evaluate the condition of cells after exposure and to localize metal ions at the sub‐cellular level. Predictably, metal treatment exhibited toxicity at selected concentrations by suppressing the growth of *Nitratiruptor* sp. SB155‐2 (Figure 1A) and inducing distinguishable transcriptomic responses to Cd(II) and Cu(II). When so treated, 385 (19.3%) and 629 (31.6%) genes were differentially transcribed in the presence of the two metals, respectively (Figures 1B,C and 2). A more notable response triggered by Cu(II) stress might be related to the essential functions this metal serves in organisms. Cu(II) is required for cell viability, but it is also highly toxic at relatively low concentrations in the cytoplasm, as opposed to nickel, zinc, and manganese, requiring multiple layers of regulatory and protein‐coding pathways to ensure cell homeostasis (Arguello et al., 2013; Bondarczuk & Piotrowska‐Seget, 2013; Rademacher & Masepohl, 2012). The response to Cu(II) appears more complex than to the non‐bioessential Cd(II), including a larger number of high‐affinity metal transporting systems, as well as oxidative stress‐responsive genes (Tables S3 and S4). Additionally, sulfur, cysteine, and methionine metabolic pathways were up‐regulated only under Cu(II) stress, while chemotaxis and flagella formation were up‐regulated only under Cd(II) stress (Table 2, Figure 3). Up‐regulation of these pathways was confirmed by proteomic analysis of proteins involved in bacterial chemotaxis, which were significantly more abundant under Cd(II) stress, whereas proteins related to the cysteine and methionine pathways were significantly more abundant under Cu(II) stress. Several other proteins involved in sulfur metabolism and oxidative stress were also up‐regulated in metal‐treated samples (Figure 5). The various patterns in transcriptomic and proteomic responses are consistent with our observations using electron microscopy and EDX. While pPLGs were present regardless of culture conditions, their role in metal sequestration varied, depending on the metal (Figure 6). While the Cd(II) content of pPLGs was relatively high, a possible sequestration strategy for Cd(II), the role of pPLGs under Cu(II) stress remains uncertain. In some cells, EDX analysis of pPLGs showed relatively higher levels of Cu(II) than in control or Cd‐treated cells, while in others, there were no visible differences between treatments (Figure 7). Additionally, Cd(II) exposure triggered formation of CdS precipitates (Figure 8) as a Cd(II) detoxification strategy. The current results shed new light on Cd‐ and Cu‐responsive molecular mechanisms of a deep‐sea hydrothermal vent bacterium of the Phylum Campylobacterota.

### Common genetic strategies against Cd(II) and Cu(II) stress

Bacteria have developed efficient metal‐specific adaptations (Hu et al., 2005; Lu et al., 2017; Villagrasa et al., 2021), and accordingly, *Nitatiruptor* sp. SB155‐2's transcriptomic profile differs markedly when responding to Cd(II) or Cu(II) (Figure 2). Among the 27 commonly up‐regulated DEGs, a large proportion are likely involved in metal efflux complexes (Nakagawa et al., 2007).

The first of two gene clusters, encoding multicomponent transport systems, presumably correspond to the ars operon, a conserved arsenic (As) detoxification mechanism in gram‐negative bacteria (Diorio et al., 1995). Arsenic resistance in bacteria is mediated by the arsRBC genes, known as the *ars* operon. arsR encodes an arsenic ion repressor that regulates expression of an arsenate reductase (arsC), which transforms As (V) into As (III) and an inner membrane‐associated As (III) export system (arsB) responsible for export of As (III) from the cell (Moore et al., 2005). Previous studies have found that transcription of arsR is less metal‐specific than expected, given that it is also induced by other metals, such as zinc (Peng et al., 2018), cadmium (Hu et al., 2005; Moore et al., 2005), antimony, or bismuth (Hu et al., 2005; Moore et al., 2005), initiating subsequent up‐regulation of the *ars* operon. One possible explanation for induction of the *ars* operon following Cd(II) exposure is the similarity of the metal‐binding site of arsR to that of cadC, the metal‐binding repressor for the *cad* operon and specific for Cd(II) (Bairoch, 1993; Yoon & Silver, 1991). Nevertheless, metal exposure experiments using *Cupriavidus metallidurans* CH34 revealed that other metals, including copper, cobalt, or nickel, failed to relieve repression of arsR (Zhang et al., 2009), contrary to our observations for Cu(II).

As for the second cluster, the SO_0444 family Cu/Zn efflux transporter seems to be equally induced by Cu(II) and Zn, according to its annotation. In non‐specific induction, Cd(II) can replace zinc in zinc‐requiring proteins, including efflux transport systems, as both elements share chemical and physical properties (Tang et al., 2014). TolC family proteins are very versatile outer membrane channels (OMC) involved in export of compounds of different sizes and compositions (Federici et al., 2004; Tanabe et al., 2009; Turlin et al., 2014). This protein can function in combination with other efflux pumps, such as RND, ABC, or MFS, suggesting that this operon may also belong to one of these large families of efflux pumps, even though BLAST searches did not provide further hints about it. Since SO_0444 family Cu/Zn efflux transporter seems to be central to Cd(II) and Cu(II) detoxification by *Nitratiruptor* sp. SB155‐2, better characterization of this operon is needed in future studies.

Excessive cytosolic metal concentrations produce oxidative stress, threatening cell homeostasis. One of the main consequences is the loss of protein structure and function due to protein unfolding and misfolding (Imamoglu et al., 2020). In such cases, different chaperone systems, such as the well‐known DnaK/DnaJ and GroES/GroEL systems, are rapidly and efficiently activated (Susin et al., 2006), and as expected, genes encoding both chaperone systems were up‐regulated by both metals in *Nitratiruptor* sp. SB155‐2. Also, glutamate 5‐kinase is likely involved in stress alleviation. This enzyme catalyses the transfer of a phosphate group to glutamate to form l‐glutamate 5‐phosphate and it is involved in synthesis of proline (Pérez‐Arellano et al., 2006). This amino acid serves several protective functions as an osmoprotectant or scavenger of reactive oxygen species (ROS) (Meena et al., 2019); hence, it facilitates adaptation to environmental stress in bacteria (Siripornadulsil et al., 2002). For instance, Al‐Mailem et al. (2018) found that the addition of proline to hypersaline soil containing heavy metals enhanced heavy metal tolerance in four halophilic and hydrocarbonoclastic bacteria and proved useful for bioremediation. Additionally, protein oxidation, as a consequence of oxidative stress, results in proteolytic degradation. Serine proteases have proven especially important for bacterial survival under multiple stress conditions (Zarzecka et al., 2019). The gene encoding a DegQ family serine endoprotease was highly up‐regulated, and significant levels of this protein were also found under Cd(II) treatment, indicating that it is strongly induced under metal stress, making this gene a putative biomarker for metal stress (Song et al., 2020).

### Cadmium stress induces flagella formation and CdS production as major tolerance/detoxification mechanisms in *Nitratiruptor* sp. SB155‐2

The genetic determinants, *czc* and *cad*, are major efflux systems that confer Cd(II) resistance in bacteria. In addition to Cd(II), the protein complex, czcCBA, a member of the RND family, can also export cobalt, zinc, and nickel in a number of bacterial species, for example, *Pseudomonas aeruginosa* (Ducret et al., 2020), *Escherichia coli* (Nies, 1995), *Ralstonia metallidurans* (Nies, 2003), and *Pseudamonas putida* (Peng et al., 2018). The *Nitratiruptor* sp. SB155‐2 genome includes one candidate gene for CzcB (NIS_RS03660) and another for CzcA (NIS_RS04945), whereas no gene candidates were found for CzcC. As a second line of defence, the P‐type ATPase, CadA, also contributes to Cd(II) tolerance, being the primary determinant of Cd(II) resistance in species like *Bacillus subtilis* (Moore et al., 2005) or *Staphylococcus aureus* (Nucifora et al., 1989). Genomic analysis of *Nitratiruptor* SB155‐2 also confirmed the presence of a candidate gene encoding CadA P‐type ATPase (NIS_RS07760). Unexpectedly, our transcriptomic results show that Cd(II) stress did not up‐regulate any of the genes encoding these two Cd‐specific efflux transport systems (Table S3). The ATP‐binding cassette (ABC) family, one of the largest transporter families, is involved in both import and export of substances, including toxic substances and can also contribute to metal homeostasis (Ma et al., 2009). Our results suggest that few ABC transporters likely contribute to Cd(II) resistance (Table S3).

Chemotaxis and flagella formation were over‐represented pathways under Cd(II) stress (Figures 3 and 5, Table 2, Table S6). It seems reasonable to think that *Nitratiruptor* sp. SB155‐2 cells can respond to toxic levels of Cd(II) by activating flagella formation and chemical sensing in order to find more favourable conditions. By activating these pathways, bacteria can move efficiently in response to steep, fast‐changing gradient fluctuations in vent environments. In other gram‐negative models, flagella formation under Cd(II) stress causes opposing responses. Some species of bacteria lost mobility due to loss of flagella (Siripornadulsil et al., 2014). In others, transcriptomic profiles show a clear up‐regulation of genes encoding flagellar proteins as a primary response to Cd(II) (Ma & Sun, 2021). The presence of this appendage would enable bacterial cells to find favourable environmental conditions for growth and survival in vent environments (Matilla & Krell, 2018).

Biomineralization of metals is an efficient strategy for reducing metal presence in cytosol. The production of CdS particles by bacteria growing under high Cd(II) concentrations has been observed in other prokaryotes, including the deep‐sea hydrothermal vent bacterium, *Idiomarina* sp. OT37‐5b (Ma et al., 2020) and *Pseudoalteromonas* sp. MT33b (Ma & Sun, 2021). These authors found that methionine gamma‐lyase was the main enzyme involved in formation of CdS particles through desulfurization of cysteine. The *Nitratiruptor* sp. SB155‐2 genome does not possess a gene encoding a methionine gamma‐lyase, but BLAST searches revealed two O‐acetylhomoserine aminocarboxypropyltransferases (NIS_RS06840 and NIS_RS06845, not significant and *p*adj < 0.05, respectively) under Cd(II) stress. These were highly similar to the *Idiomarina* sp. OT37‐5b methionine gamma‐lyase, which may be involved in formation of CdS particles. Bacteria inhabiting vent environments could obtain cysteine from environmental sources, in the form of organo‐sulfur molecules (OMS) (Ma et al., 2020). Organo‐sulfur molecules contribute to sulfur cycling in hydrothermal vent environments, but bacterial uptake mechanisms are unknown (Wasmund et al., 2017).

### *Nitratiruptor* sp. SB155‐2 relies on efficient efflux and cysteine and methionine metabolism for Cu(II) detoxification

The three main genetic determinants of Cu resistance in bacteria, *cus*, *cop*, and *cue* (Franke et al., 2003), are present in the *Nitratiruptor* sp. SB155‐2 genome. In bacteria, the RND complex, cusCFBA, is involved in direct export of periplasmic Cu out of the cell (Franke et al., 2001; Nies, 2003). While we found candidate genes for CusA and CusB, *Nitratiruptor* sp. SB155‐2 was devoid of candidate genes encoding CusC and CusF. Contrary to expectations, neither CusA nor CusB was differentially transcribed under Cu(II) stress, suggesting that *cus* may not be essential for Cu(II) detoxification in these bacteria, at least, under the micro‐aerobic conditions in this study. Activity of *cus* was dominant under anaerobic conditions (Outten et al., 2001), while the heavy metal translocator P‐type ATPase, CopA, in combination with the periplasmic multicopper oxidase (CueO) usually operates under aerobic conditions (Outten et al., 2001). The main mechanism of the CopA‐CueO export system involves Cu(II) export by CopA from the cytoplasm to the periplasm, where it is oxidized by CueO from Cu(I) to Cu(II) to protect periplasmic enzymes from copper‐induced damage (Grass & Rensing, 2001). CopA and CueO were significantly up‐regulated exclusively in samples treated with Cu(II) (Tables S3 and S4), suggesting that the combination CopA‐CueO is a major mechanism for Cu(II) detoxification in *Nitratiruptor* sp. SB155‐2.

Gene enrichment and proteomic analysis suggest that sulfur may play a central role in Cu(II) resistance by *Nitratiruptor* sp. SB155‐2. Periplasmic proteins enriched in the sulfur amino acids, methionine and cysteine, bind Cu(II) with high affinity. Other studies of gram‐negative bacteria (Franke et al., 2003; Long et al., 2010; Lu et al., 2017) confirm that methionine residues are essential for Cu binding in the main Cu‐specific transport systems. Crystal structure analysis of the inner membrane transporter, CusA, revealed four methionine pairs, in addition to the three methionine metal‐binding sites located in the cleft of the periplasmic domain (Long et al., 2010). Because of this structure, CusA is capable of binding Cu directly from the cytosol and periplasm using methionine clusters (Su et al., 2011). Similarly, CopA, a methionine‐rich periplasmic protein, is able to bind up to 11 Cu atoms (Puig et al., 2002). Other examples include periplasmic proteins, PcoC or CopC, methionine‐rich proteins able to bind both Cu(I) and Cu(II) (Bondarczuk & Piotrowska‐Seget, 2013; Lawton et al., 2016; Roberts et al., 2002; Wernimont et al., 2003). Other than its incorporation into periplasmic transport proteins, methionine is required for synthesis of glutathione, a universal antioxidant, in response to heavy metal‐induced oxidative stress (Stewart et al., 2020). Glutathione synthase is exclusively up‐regulated in Cu‐treated samples (Table S4).

### pPLGs serve different functions under Cd(II) and Cu(II) stress

Two contrasting functions have been described for pPLGs in the presence of toxic metals: (1) the number of pPLGs per cell increases in order to sequester excess metal (Keasling & Hupf, 1996; Villagrasa et al., 2021); (2) pPLGs are hydrolyzed in order to transport metal ions towards the periplasmic space bound to inorganic phosphate (Alvarez & Jerez, 2004; Seufferheld et al., 2008). Our results suggest that *Nitratiruptor* sp. SB155‐2 adopts the first strategy for metal sequestration. Unlike eukaryotes, bacteria do not have discrete cellular compartments or organelles; thus, metal‐binding metabolites, as well as storage and sequestration mechanisms, are crucial in order to keep metal concentrations under control (Chandrangsu et al., 2017). Among many other roles, pPLGs are involved in metal chelation, especially in bacteria that do not harbour superoxide dismutase (SOD), a highly conserved enzyme for detoxification of superoxide anion (Docampo, 2006). The *Nitratiruptor* sp. SB155‐2 genome analysis revealed an absence of SOD (Nakagawa et al., 2007), implying that pPLGs serve as the primary sequestration mechanism in these bacteria. However, in *Nitratiruptor* sp. SB155‐2 the function of pPLGs in sequestration may be somewhat Cd‐specific when compared to Cu(II) exposure. While the reasons for an increased number of pPLGs under Cu(II) stress remain unknown, its relatively low concentration compared to Cd(II), may be due to the Cu contribution to various biological processes, as well as to the presence of efficient efflux mechanisms, which closely regulate intracellular Cu(II) concentrations. Additionally, the number and size of pPLGs in this study should be interpreted with caution, since more precise microscopic techniques should be used to evaluate these features accurately, such as with FIB‐SEMS, since 2D micrographs can only provide an incomplete picture of these 3D structures.

Mobilization of pPLGs in bacteria is performed by the enzymes, polyphosphate kinase (PPK) and exopolyphosphatase (PPX), which synthesize and hydrolyze pPLGs, respectively. The *Nitratiruptor* sp. SB155‐2 genome possesses two PPKs and two candidate PPXs. Surprisingly, neither PPK nor PPX was differentially transcribed under conditions tested in this study. The lack of differential expression for these genes could be a result of a mismatch in the sampling timing for RNA extractions vs expression time required for these genes. Previous studies on the metal‐resistant archaeon, *Metallosphaera sedula*, showed that a transcriptional shift of PPX occurred only 30 min after Cu(II) exposure, and after 3 h, PPX levels were again similar to those of controls (Rivero et al., 2018). In order to fully understand the transcriptional shift of these enzymes in *Nitratiruptor* sp. SB155‐2 following metal exposure, future characterization should include a fine‐scale time‐course experiment to study expression level dynamics of these genes. Together with pPLGs, the periplasmic space of gram‐negative bacteria can be used as an intracellular compartment providing a storage or detoxification area to keep metal ions out of the cytosol (Ma et al., 2009).

Finally, even though pPLGs and other subcellular structures are well known in bacteria, application of modern high‐resolution microscopy techniques in combination with high‐throughput sequencing approaches can provide novel details regarding the timing and specific roles of these structures under metal stress. Future studies may benefit from these technologies to better understand detailed mechanisms of these structures regarding metal tolerance in microbes associated with deep‐sea hydrothermal ecosystems. Prokaryotes inhabiting metal‐enriched niches will reveal new metabolic capabilities and metal detoxification solutions (Antwis et al., 2017). These mechanisms could contribute significantly to new approaches for environmental restoration and remediation.

## AUTHOR CONTRIBUTIONS

A.A., T.N, S.S., designed the experiments. A.A., S.S., T.S, S.S performed the experiments. A.A., S.M., analysed the data. A.A. drafted the article with inputs from all the authors. All the authors aproved the submitted version.

## CONFLICTS OF INTEREST

All authors declare that they have no conflicts of interest.

## Supporting information


**Appendix S1:** Supplementary Information
